# Multi-Source Ensemble Learning for the Remote Prediction of Parkinson's Disease in the Presence of Source-Wise Missing Data

**DOI:** 10.1109/TBME.2018.2873252

**Published:** 2018-11-05

**Authors:** John Prince, Fernando Andreotti, Maarten De Vos

**Affiliations:** 1 Department of Engineering SciencesInstitute of Biomedical Engineering, University of Oxford 473881 OxfordOX1 2JDU.K.; 2 Department of Engineering SciencesInstitute of Biomedical Engineering, University of Oxford 473881

**Keywords:** Missing data, Parkinson's disease, multi-source learning, convolutional neural networks, ensemble learning, feature selection, bootstrap statistics, mobile-Health

## Abstract

As the collection of mobile health data becomes pervasive, missing data can make large portions of datasets inaccessible for analysis. Missing data has shown particularly problematic for remotely diagnosing and monitoring Parkinson's disease (PD) using smartphones. This contribution presents multi-source ensemble learning, a methodology which combines dataset deconstruction with ensemble learning and enables participants with incomplete data (i.e., where not all sensor data is available) to be included in the training of machine learning models and achieves a 100% participant retention rate. We demonstrate the proposed method on a cohort of 1513 participants, 91.2% of which contributed incomplete data in tapping, gait, voice, and/or memory tests. The use of multi-source ensemble learning, alongside convolutional neural networks (CNNs) capitalizing on the amount of available data, increases PD classification accuracy from 73.1% to 82.0% as compared to traditional techniques. The increase in accuracy is found to be partly caused by the use of multi-channel CNNs and partly caused by developing models using the large cohort of participants. Furthermore, through bootstrap sampling we reveal that feature selection is better performed on a large cohort of participants with incomplete data than on a small number of participants with complete data. The proposed method is applicable to a wide range of wearable/remote monitoring datasets that suffer from missing data and contributes to improving the ability to remotely monitor PD via revealing novel methods of accounting for symptom heterogeneity.

## I. Introduction

Parkinson's disease (PD) is the second most common neurodegerative disease after Alzheimer's disease and its prevalence is estimated to double over the next two decades [22]. Correct and timely diagnosis of PD is essential in order to properly treat symptoms prior to deterioration whilst also alleviating the long-term financial burden the disease management places on healthcare systems worldwide [7].

The current gold-standard of diagnosing and monitoring PD is the Unified Parkinsons Disease Rating Scale (UPDRS) which is performed in-clinic by movement disorder specialists [9]. However, the UPDRS is widely known to suffer from both inter- and intra-rater subjectivity often leading to high levels of false-positive diagnoses [1], [11]. The diagnosis procedure is further complicated as symptom prevalence is highly heterogeneous in the PD population in that two people with similar UPDRS scores may exhibit different motor symptoms [21], [29].

Many studies have shown the ability to identify disease differentiating digital biomarkers in gait, dexterity, tremor, and voice tests [6], [16], [24], [36]. Unfortunately, the vast majority of these studies have been performed in-clinic, using different experimental protocols, different sensors, and have had small cohorts. Indeed, a recent review found that 77% of such studies had a cohort of under 30 participants [29]. As such, the specific biomarkers from each study lack scalability as they are yet to be validated on a large cohort.

Due to the subjectivity of the UPDRS system and limited data of current studies, the use of wearable sensors to identify digital biomarkers that are capable of objective disease quantification are now widely being sought [19], [31]. The use of smartphones to monitor PD in a non-clinical environment overcomes many of the challenges of PD quantification. Smart-phones contains many sensor types (accelerometer, touchscreen, microphone) therefore afford the opportunity of extracting digital biomarkers relating to multiple motor symptoms. Additionally, tests are able to be completed on a daily basis without the requirement of going to a hospital.

Several observational studies have been initiated using smartphones for data collection and have yielded ‘big’ datasets from cohorts of far greater sizes [3], [39]. These studies allow volunteers to download a smartphone application and contribute measurement data in multiple test types (gait, dexterity, voice, and tremor) and demographic data including symptom severity. However, these datasets suffer from many sources of noise that has restricted their large-scale findings from being considered clinically relevant [34]. As the tests are completed in remote environments they often suffer from the influence of inconsistent environmental conditions and improper test completion. As such, disease classification accuracy using data collected from remotely collected datasets has been dramatically lower than when the same tests are completed in a clinical-environment [20]. It has been recommended that more sophisticated machine learning techniques be applied to larger cohorts in a bid to improve remote classification results.

The largest limitation of smartphone based datasets is the large quantity of missing data and poor volunteer retention rates. As participants are able to contribute data in multiple test types, it is commonly found that the vast majority of participants only complete a subset of tests [20], [26], [40]. Due to the heterogeneous nature of PD symptoms, the research community is particularly interested in determining the relationship between the different motor symptoms. When a dataset contains missing tests from many participants, it is commonly called a *source-wise* missing dataset, where the terms *source* and *test* are interchangeable. Source-wise missing datasets commonly occur and are especially prominent in datasets being collected in remote environments [2], [39].

Imputation techniques are commonly employed in the case of missing data [25]. However, in the case of source-wise missing medical data, imputation is highly inappropriate. If a source is missing then the complete set of associated features is missing, therefore, a large number of features must be imputed from other sources which may contain no mutual information to the missing source, resulting in the imputations being poorly representative of their intended values [38]. An alternative approach in source-wise missing data is to discard all observations with incomplete data [2], [20]. Although this guarantees a complete dataset, it is wasteful of potentially relevant data, especially if a large number of observations have incomplete data. In a recent study of remotely monitoring PD, 48.4% of the available participants were discarded from the study due to incomplete source data [40].

In this research we present a method to compensate for source-wise missing data via combining a dataset deconstruction technique with ensemble learning. We demonstrate how a data retention rate of 100% can be achieved even if a majority of observations have incomplete data. We apply our method to the large, remotely collected, and mostly incomplete dataset for the purpose of PD classification. We compare the classification ability of our method to that of the current means of compensating for source-wise missing data and show that the inclusion of additional data can improve classification and feature selection. We further illustrate the potential of learning deep convolutional neural network classifiers for this application. This became only possible because of the size of the dataset, which is an order of magnitude larger than the classical “complete” datasets.

## II. Materials and Methods

### A. Dataset Description

The data used throughout this research was collected as part of the mPower study [10]. Healthy controls (HCs) and PD subjects enrolled into the study via downloading and providing consent through an iPhone (Apple Inc., Cuppertino, USA) application. Due to PD being most prominent in people over the age of 50 years old, we only include PD and HC participants 50 years or older in this analysis. Moreover, since the participant retention rate is low in the mPower study, in this work we focus in the data provided 24 hours following the completion of their first source, resulting in 1,513 subjects [26]. The mPower application allowed participants to complete regular self-assessed severity surveys and a one-off demographics survey in addition to four different activities intended to test for the presence or severity of PD.

The *tapping activity* takes the form of the alternate finger tapping (AFT) test which is commonly used in-clinic to assess dexterity [22]. This activity requested the participant place their iPhone on a flat surface and alternately tap two on-screen buttons using two fingers for 20 seconds. In the *walking activity* participants were asked to place their iPhone in their front trouser pocket and to walk in a straight line for 20 steps, turn around, and then walk back along the same route. The *voice activity* asked participants to make a sustained /a/ (‘Aaaaah’) phonation into their iPhone microphone for 10 seconds. The *memory activity* was the only activity intended to assess non-motor characteristics of PD. During the memory activity, a grid of flowers appeared on the iPhone screen and a number of flowers were illuminated in a random pattern. Participants were asked to recall the pattern in which the flowers were illuminated. During each activity instance this process was repeated for up to five levels with the grid of flowers and the number being illuminated at each level increasing. During these activities tri-axial accelerometer and gyroscope, touchscreen (for tapping) and microphone data (voice test) were collected.

### B. Dataset Deconstruction and Model Framework

**Fig. 1.**
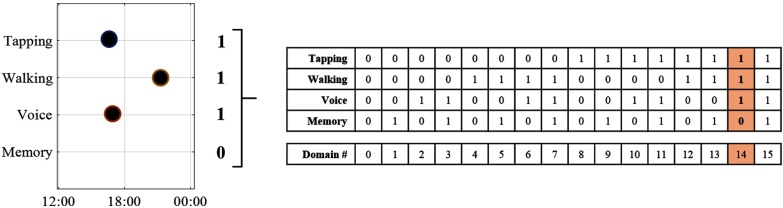
The domain assignment procedure. A participant has contributed a specific combination of the four sources. This combination of sources is represented as a binary vector and the participant is subsequently assigned to the decimal integer domain which the binary vector represents as highlighted in red.

Individual participants were able to contribute with any of the $S$ sources (here $S=4$: tapping, walking, voice, and memory) in any given combination, thus the database comprises $2^{S}$ possible combination of the available tests. Each different combination of sources may be represented as a binary vector, $\mathbf {I}[1\ldots S]$, where $\mathbf {I}[i] = 1$ demonstrates that the $i\text{th}$ source has been contributed. In this research, a participant is assigned a binary source vector based on which sources were contributed. The binary source vector determines which *domain* a participant belongs to. A demonstration of the domain assignment process for a participant in the mPower dataset is given in Fig. 1. Via assigning each participant to a domain, we have subsequently partitioned the initial source-wise missing dataset into $2^{S}$ smaller but complete domains; a process herein called *dataset deconstruction*. A degree of overlap exists with regards to which sources are present between certain domains. For example, in Fig. 1 it is evident that domains 7, 13, and 15 contain the same sources (in addition to at least one other source) as present in domain 5. The ability for data representation to be shared across domains presents the opportunity to apply multi-task learning (M-TL) wherein multiple learning tasks are solved simultaneously [28]. Indeed, M-TL has been applied using this dataset deconstruction technique but there are several limitations to its implementation [38]. The first limitation is that the number of participants in each domain is inconsistent which results in the number of participants used to train and test each of the M-TL models also being highly inconsistent. Thus, the results of a M-TL model when tested on each domain could be confounded by (i) the number of participants in the domain (ii) the characteristics of these participants, or (iii) the sources present each domain. These confounding factors make the M-TL results difficult to interpret.

To overcome these limitations, we note two special cases of the dataset deconstruction. Firstly, we focus on the *single source dofmains* where only a single source is present; in the mPower dataset these are domains 1, 2, 4, and 8 which correspond to the memory, voice, walking, and tapping individual source domains respectively. For each single source domain, all participants who contributed the source are eligible to be used to develop the individual source model, regardless of which domain they have been assigned to. The example participant in Fig. 1 would therefore be eligible to develop models for the tapping, walking, and voice single source domains. Subsequently, even participants with incomplete source data can be used in developing the individual source models thus ensuring a 100% participant retention rate. A participant with missing data who completed $n$ sources ($0 < n \leq S$) is eligible to be used in developing $n$ individual source models. Secondly, we note that the participants with complete source data (domain 15) are the only participants eligible to be used in developing *all* of the individual source models. Consequently, participants with complete source data will be assigned as a test group against which all individual source models will be tested. Furthermore, the individual source models can be fused into all possible combinations through the use of source ensembles. All models created through source ensembles can also be tested on the participants with complete source data.

We can now formally define our model framework. Via deconstructing the source-wise missing dataset, we are able to develop $S$ individual source models using all participants, even those with missing data. The participants with complete source data are excluded from the training/validation of the individual source models and are reserved to act as a test set against which all individual source models, and their $2^{S}$ combinations, may be tested. Having created a consistent test set for all models, the results of all models will be directly comparable to one another, thus removing the aforementioned confounding factors. In the mPower dataset, we identified 1,380 participants with incomplete source data who form the training and validation set. The instances and demographics of the training and validation participants are given in Table I. We identified 133 participants (87 PD/ 46 HC, age 62.9 $\pm$ 7.6, 71% Male) with complete source data who are assigned as the test set. Each participant in the test set contributed one instance in each of the sources. The effects of sex are assumed to be negligible on test performance as has been previously suggested on the mPower dataset [5], [26].

**TABLE I:** Demographic Data of Participants With Incomplete Data Contributions Who Form the Training and Validation Set for Each Source

	Tapping	Walking	Voice	Memory
# Of Parts. (PD/HC)	1323 (799/524)	624 (377/247)	1072 (679/393)	39 (25/14)
Age	62.4 $\pm$ 7.7	62.8 $\pm$ 7.6	62.9 $\pm$ 7.7	62.6 $\pm$ 8.7
% Male	68.6	71.2	67.8	61.5

### C. Individual Source Model Development

#### 1) Feature Based Classifiers

We utilized three classifiers that require an explicit feature set to be extracted in order to develop the individual source models. From the tapping activity we extracted features from the raw touchscreen data and from the corresponding accelerometer waveforms. The touchscreen features pertain to rhythm, spatial variability, and fatigue whilst a widely used set of signal based features are extracted from the accelerometer waveforms [18], [39].

From the walking activity we extracted the same set of signal based features as in the tapping activity from the tri-axial accelerometer and gyroscope. As the raw waveforms are known to contain segments of noise or periods of no movement, features were only extracted from sections of the signal that were identified as gait as determined by a gait-segmentation algorithm [4].

Features were extracted from the voice recordings using the publicly available Matlab (Mathworks, USA) toolbox developed by [37]. These features utilize both temporal and frequency based metrics and have proven capable of detecting dysphonic PD participants in remote environments [36].

The memory activity provided only three features; total score, number of levels attempted, and number of incorrect responses.

The tapping, walking, voice, and memory activities provided 97, 180, 326, and 3 features respectively. We developed three feature based classifiers for each individual source. Two of these classifiers are commonplace in the prediction of PD; logistic regression (LR) and random forests (RF). We also implement a state-of-the-art Deep Neural Network (DNN) on this feature set. All DNNs consisted of five layers with 200, 300, 50, 32, and 1 layer respectively. All activation functions were rectified linear units (ReLU) due to their ability to improve training time whilst avoiding the vanishing gradient problem [13], [17].

During the training and validation of the LR models, we implemented least absolute shrinkage and selection operator (LASSO) feature selection on the training data:

\begin{equation*} \arg \min _{\beta } \frac{1}{2} ||\mathbf {y} - \mathbf {X} \boldsymbol{\beta } ||^{2}_{2} + \lambda ||\beta ||_{1} \tag{1} \end{equation*}

where $\mathbf {X}$ is the design matrix, $||\cdot ||_{1}$ is the the $\ell _{1}$-norm regularization term which induces sparsity within $\beta$, and $\lambda _{1}$ is the regularization coefficient. The resulting dense feature set (all features whose $\beta \ne 0$) is extracted from the validation set and used for prediction.

#### 2) Convolutional Neural Network Classifier

In addition to the three feature based base classifiers, we also implement Convolutional Neural Networks (CNNs). CNNs are widely considered to be the state-of-the-art machine learning techniques and have received little attention in the field of PD classification to date due to the generally small size of PD datasets [27]. CNNs do not require the definition of an explicit feature set but rather are capable of automatically learning features, or *filters*, directly from raw data. Furthermore, these filters are translationally invariant making CNNs particularly well suited to noisy raw data as in the mPower database.

Of the four activities in the mPower database, the tapping, walking, and voice activities present data suitable to be used as inputs to a CNN (i.e. raw time series). In the tapping activity, we use the raw tri-axial accelerometer waveforms (sampling frequency *fs* = 100 Hz) alongside the touchscreen pixel coordinate data. As the touchscreen data is unevenly sampled we use linear interpolation to create waveforms of equal length to the accelerometer waveforms [27]. In the walking activity the tri-axial accelerometer and tri-axial gyroscope signals were used as the raw input (*fs* = 100 Hz). In the voice activity the raw voice recording signal was used (*fs* = 42 kHz). All waveforms, excluding the interpolated tapping touchscreen waveform, underwent zero-mean unit-variance normalization.

**Fig. 2.**
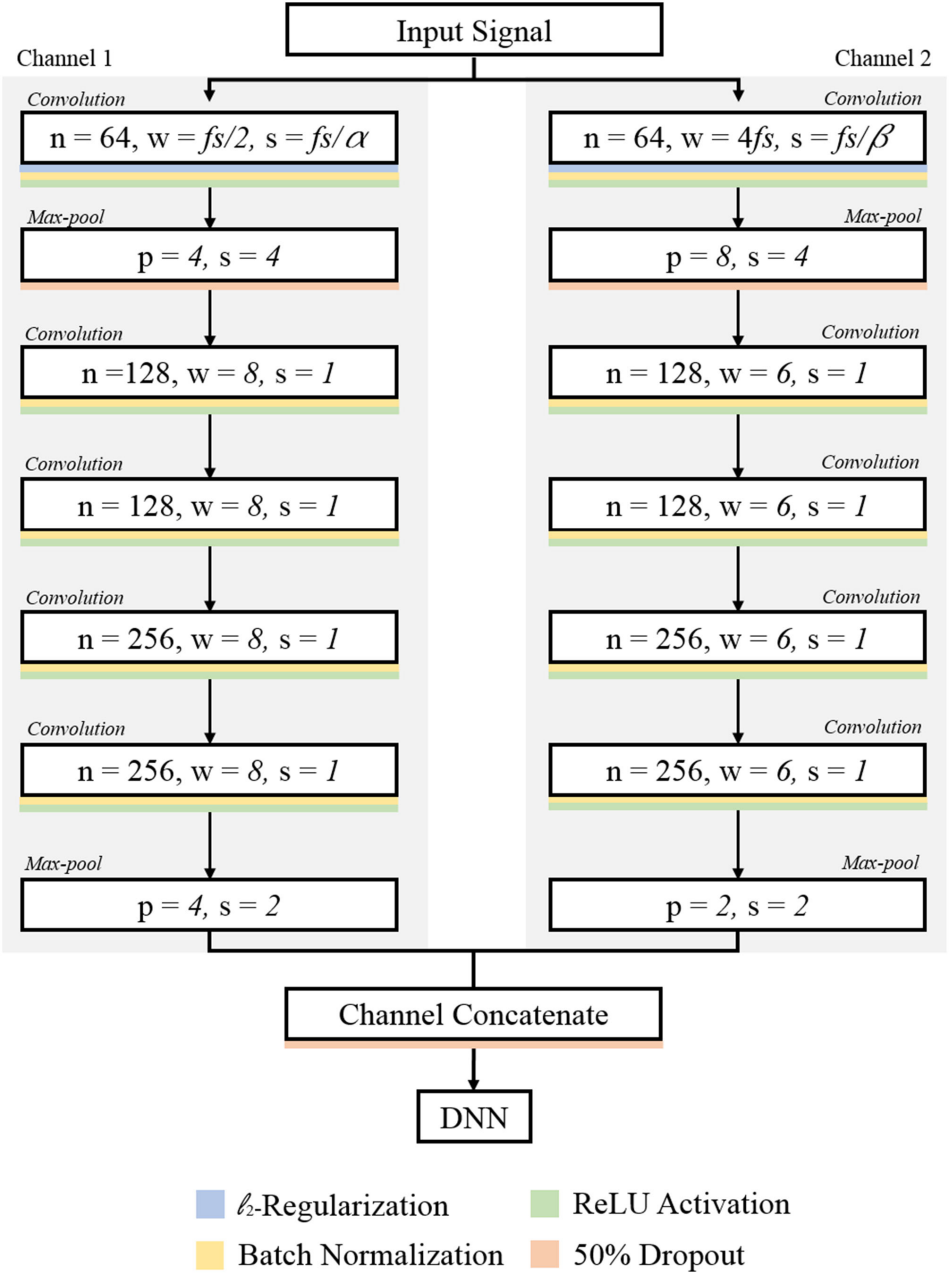
The multi-channel CNN architecture used on the voice, walking, and tapping source data. Here *fs* is the signal sampling frequency, *n* is the number of filters, *w* is the width of the filters, *s* is the stride length of convolutions, and *p* max-pool size.

The architecture of the multi-channel CNN used on all activity types is shown in Fig. 2. While additional benefit might be obtained by using different network structures for the different sources, we opted for a generic general framework. Here, the concept of a variable first receptive field width is exploited across the two convolutional branches [33]. When using convolutional filters of a large width, the frequency components of the data are better captured. Conversely, using filters of a small width better capture temporal aspects of the signal. Thus, the width of the first convolutional filter in each of the two channels is different so as to capture both temporal and frequency components of the data. Alternative CNN architectures use small receptive fields but require many more layers and convolutional operations in order to capture the frequency components of the data [10], [35]. Our architecture utilises $\ell _{2}$-norm regularization, max-pooling, and batch normalisation layers as extra means of preventing overfitting, parameter reduction, and reduce training time respectively.

All CNN and DNN networks were developed using using Keras with a Tensorflow (Google Inc., California) back-end using *Adam* optimization and model loss was calculated through binary cross-entropy [12]. All neural network models were implemented using a Dell (Dell Inc., TX) T640s computer with 256GB of RAM and a NVIDIA (Santa Clara, CA) 1080 TI GPU. The training run-time of the CNN models ranged from six (tapping and walking) to ten hours (voice). The python scripts used to train and validate all CNN models are available at the authors GitHib repository (www.github.com/johnPrinceOx/Multi-Source_Ensemble_Learning_Neural_Networks).

#### 3) Individual Source Model Evaluation

The classifiers developed for each individual source were trained and validated using the individual source domains (Table I). During the training and validation of each classifier we performed repeated stratified 10-fold cross validation. Prior to being separated into folds, the data is balanced so as to have equal number of PD to HC participants. In the feature based classifiers the training and validation sets are normalized to zero mean-unit variance using the means and standard deviations of the training feature set so as to avoid data leakage. The accuracy of each classifier is reported for the training and validation set. As the test set is imbalanced, when the individual source models are applied to the test set we also report the $F_{1}$-score (harmonic average) in addition to the classification accuracy.

### D. Ensemble Learning Approaches

Ensemble learning, often called classifier fusion, enables the predictions of multiple algorithms to be fused into a single prediction [23]. Often, the single ensemble prediction outperforms each of the ‘base’ algorithms due to the ensemble accounting for variability within the base algorithms prediction ability [15].

We present two forms of ensemble learning (classifier and source) which enable two types of variability to be accounted for simultaneously.

#### 1) Classifier Ensemble

Via performing a classifier ensemble within each individual source, we assessed whether the classification ability of an ensemble of multiple classifiers outperforms each individual base classifier.

We use two popular classifier ensemble learning algorithms: majority voting and mean probability [14], [30]. Suppose we would like to ensemble the responses of $B$ base classifiers for a single observation, we denote an ensemble learner $\mathcal {F}$ as:

\begin{equation*} \mathcal {R} = \mathcal {F}(R_{1}, \ldots ,R_{B}) \tag{2} \end{equation*}

where $R_{i}$ is the response of the $i\text{th}$ classifier and $\mathcal {R}$ is the ensemble response. Majority voting is implemented on the binary responses of each classifier and is defined as:

\begin{equation*} \mathcal {F} = {\begin{cases}1 & \text{if } \sum ^{B}_{i=1} R_{i} \geq \frac{B}{2} \\ 0 & \text{if } \sum ^{B}_{i=1} R_{i} < \frac{B}{2} \end{cases}} \tag{3} \end{equation*}

Majority voting therefore returns the binary response that occurs most frequently between the base classifiers for a single observation. The mean probability ensemble is performed on the ‘soft’ response of each classifier and is defined as:

\begin{equation*} \mathcal {F} = {\begin{cases}1 & \text{if } \frac{\sum ^{B}_{i=1} P(R_{i}=1)}{B} \geq 0.5 \\ 0 & \text{otherwise} \end{cases}} \tag{4} \end{equation*}

**Fig. 3.**
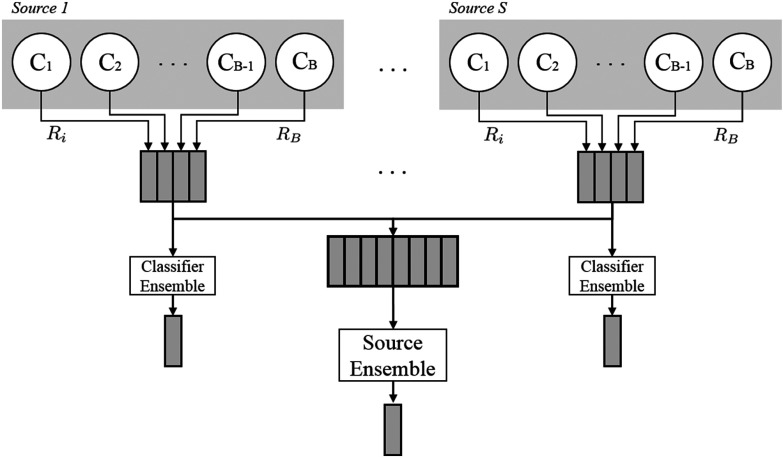
Schematic of the classifier and source ensemble procedures. There are *B* base classifiers within each source. $R_{i}$ is the response from the $i\text{th}$ classifier. A classifier ensemble is implemented on the responses from all *B* classifiers from a single source. A source ensemble is performed on all responses from all classifiers from the $S$ sources.

The mean probability ensemble therefore returns a response based on the average probability of all classifiers for a single observation. A schematic of the classifier ensemble strategy is demonstrated in Fig. 3.

The first classifier ensemble was formed using the three feature based classifiers (LR + RF + CNN). The second classifier ensemble was formed using both the feature based and the CNN classifiers (LR + RF + DNN + CNN). This enables any difference between the CNN classifier to be directly compared against a purely feature based approach. We report the accuracy and $F_{1}$-score of all classifier ensembles for each source individually.

#### 2) Multi-Source Ensemble

In addition to classifier ensembles, we also implement source ensembles. Source ensembles serve two purposes in this research. The first purpose is to account for the source-wise missing data. As we have developed models for each source individually, source ensembles allow us to fuse these individual source models in all possible combinations; allowing all individual source models to be fused and implemented on the test participants. The second purpose is to account for the heterogeneity of symptom prevalence in PD. It is common for PD to manifest itself differently across the population. Thus, a participant may show mild to severe symptoms in one source but not in any others whereas another participant of equal PD severity may show mild to severe symptoms in a different source. Through source ensembles we can consolidate our classification predictions from multiple sources into a single prediction which accounts for symptoms in all sources; thus accounting for symptom heterogeneity.

When implementing a source ensemble on all $S$ sources, and assuming that $B$ base classifiers are trained for each source, we redefine our majority voting ensemble learner as:

\begin{equation*} \mathcal {F} = {\begin{cases}1 & \text{if } \sum ^{B \times S}_{i=1} R_{i} \geq \frac{B \times S}{2} \\ 0 & \text{if } \sum ^{B \times S}_{i=1} R_{i} < \frac{B \times S}{2} \end{cases}} \tag{5} \end{equation*}

and the mean probability ensemble learner as:

\begin{equation*} \mathcal {F} = {\begin{cases}1 & \text{if } \frac{\sum ^{B \times S}_{i=1} P(R_{i}=1)}{B \times S} \geq 0.5 \\ 0 & \text{otherwise} \end{cases}} \tag{6} \end{equation*}

A schematic of the source ensemble strategy is demonstrated in Fig. 3. It is important to note that we do not perform source ensembles on the response of the classifier ensemble. Rather, we use the response of each classifier from all sources. This approach ensures the maximum amount of classifier variability is present during the source ensemble.

We firstly perform source ensembles using a single base classifier from each source; thus localising the results of source ensembles without the influence of classifier ensembles. This enables us to compare the effects of classifier and source ensembles. Finally, we perform source ensembles using multiple base classifiers from all sources. As in the pure classifier ensemble, this is performed initially using only the feature based base classifiers across all sources, followed by using all feature based and CNN base classifiers across all sources.

### E. Model Comparison Approaches

We implement two alternative approaches for multi-source classification against which multi-source ensemble learning is compared. Both alternative approaches also present a platform for assessing the importance of features from all sources.

#### 1) Complete Dataset Learning

This is the approach most commonly used in the case of incomplete multi-source datasets. We select the participants with complete source data (i.e. from the test set) and discard all participants with missing data. Classification models are developed using only the participants with complete source data. As we are also interested in which features across all sources are most pertinent in the classification procedure, we implement LASSO and Sparse-Group LASSO [32]. The latter introduces regularization at the feature and source level. When implementing LASSO during Complete Dataset Learning, the design matrix $\mathbf {X}$ in Equation 1 is:

\begin{equation*} \mathbf {X}_{c} = [\mathbf {X}_{c}^{T}, \mathbf {X}_{c}^{W}, \mathbf {X}_{c}^{V}, \mathbf {X}_{c}^{M}] \tag{7} \end{equation*}

where $\mathbf {X}_{c}^{T}, \mathbf {X}_{c}^{W}, \mathbf {X}_{c}^{V}$, and $\mathbf {X}_{c}^{M}$ are the tapping, walking, voice, and memory design matrices of the $N_{c}$ participants who contributed all sources respectively and $\mathbf {X_{c}} \in \mathbb {R}^{p \times N_{c}}$ is the complete multi-source feature matrix containing $p$ features.

The same complete design matrix ($\mathbf {X_{c}}$) is used during Sparse-Group LASSO, which is defined as:

\begin{equation*} \arg \min _{\beta } \frac{1}{2} ||\mathbf {y} - \mathbf {X} \boldsymbol{\beta } ||^{2}_{2} + \lambda _{1}||\beta ||_{1} + \lambda _{2}\sum _{i=1}^{S} \sqrt{p_{i}}||\beta _{i}||_{2} \tag{8} \end{equation*}

where $\lambda _{1}$ and $\lambda _{2}$ determine the quantity of regularization at the feature and source levels respectively and $p_{i}$ is the number of features in the $i\text{th}$ source.

We report the top 10 highest weighted features from both the LASSO and SG-LASSO methods. As this section is focused on the influence of the feature selection process, we only implement a LR model. Using the identified features from both methods, we train and validate LR models and report the classification accuracy.

#### 2) Incomplete Dataset Learning

In this approach, we implement a two-stage feature selection technique starting with the participants in each of the individual source domains. Firstly, within each of the individual source domains, we determine the highest weighted features using LASSO. Feature selection is occurring at the feature level within each individual source, we are therefore able to use the large number of participants with incomplete data. We denote the features selected by LASSO from the $i\text{th}$ individual source as $\beta ^{i}$. The non-zero weighted features are selected from each source are concatenated to give the feature weighting vector for all sources:

\begin{equation*} \beta _{ISL} = [\beta ^{T}, \beta ^{W}, \beta ^{V}, \beta ^{M}] \tag{9} \end{equation*}

From $\beta _{ISL}$, we report the features with the highest weightings. In the second step, all of the features in $\beta _{ISL}$ are selected from $\mathbf {X}_{c}$ yielding:

\begin{equation*} \mathbf {X}_{ISL} = \mathbf {X}_{c}(\beta _{ISL}) \tag{10} \end{equation*}

such that the features we have selected using the large number of participants with incomplete data have now been applied to the smaller number of participants with complete data. We conclude the second feature selection process via applying SG-LASSO on $\mathbf {X}_{ISL}$.

The highest weighted features from Complete Dataset Learning can be compared against those selected in the Incomplete Dataset Learning approach and allows us to determine if feature selection and model development is better performed at the feature or source level.

As in the Complete Dataset Learning approach, we report the top 10 highest weighted features from both the LASSO and SG-LASSO methods and report the classification accuracy of the subsequent LR models.

### F. Effects of Sample Size on Feature Confidence

To further assess the influence of performing feature selection using participants with incomplete data, we investigate the effect sample size has on feature distributions. Here we examine whether using a large sample size, as in the Incomplete Dataset Learning technique, provides a more robust platform to perform feature selection than in the traditional approaches, such as the Complete Dataset Learning technique.

We utilise bootstrap sampling on the training and validation participants, using variable sample sizes. Bootstrap sampling is a non-parametric statistical technique that enables statistical measures to be estimated from a randomly sampled subset of the data [8]. To perform the bootstrap, we randomly sample $nSamp$ participants with replacement from the $N_{i}$ participants with incomplete source data. This subset of participants is known as the bootstrap sample. The feature set contributed by the bootstrap sample, $f_{bs}$, is selected and statistical measures from each feature is calculated. Statistical measures calculated from the bootstrap sample, $\mathbb {E}(f_{bs})$, are formally referred to bootstrap statistics. The process of selecting a bootstrap sample and calculating the corresponding bootstrap statistics is repeated $B$ times. Consequently, for each bootstrap statistic, we have complied $B$ estimates of the true statistical value.

It follows that if the bootstrap sample size is small, or the original population data contains a large degree of noise, the resulting bootstrap statistics will not be representative of the true value of the statistical measure. It is for this reason that performing bootstrapping using many sample sizes is beneficial in our study of feature selection. At each sample size we can determine whether the estimated feature values from a bootstrap sample are representative of the true feature values of the entire population. This allows us to decide whether a given sample size is appropriate to undergo feature selection.

We present a visualization of how feature distributions vary with sample size via calculating the mean and standard deviation as bootstrap statistics using $B = 10,000$ bootstrap samples at each sample size.

## III. Results

### A. Individual Source Model Performances

In Table II we present the classification accuracy for each classifier in each of the individual source models. For the balanced validation set, the mean and standard deviation results are shown for the repeated 10-fold stratified cross validation. The classification accuracy and corresponding $F_{1}$ scores of these models when applied to the test set are provided in Table III.

**TABLE II:** Cross-Validation Accuracies ($\%$) of the Individual Source Models on the Training Participants. Corresponding Methods: Section II-C

	LR	RF	DNN	CNN
Memory	66.3 $\pm$ 28.3	53.9 $\pm$ 36.5	65.8 $\pm$ 25.8	n/a
Voice	62.9 $\pm$ 5.2	61.8 $\pm$ 5.6	69.7 $\pm$ 8.4	**72.5 $\pm$ 4.1**
Walking	59.5 $\pm$ 6.5	55.3 $\pm$ 6.2	68.2 $\pm$ 8.6	**72.6 $\pm$ 2.8**
Tapping	60.5 $\pm$ 4.8	61.8 $\pm$ 5.1	65.3 $\pm$ 3.1	**69.4 $\pm$ 3.5**

**TABLE III:** Results of the Individual Source Models When Implemented on the Test Participants. Corresponding Methods: Section II-C

	LR	RF	DNN	CNN
	Accuracy ($\%$)			
Memory	64.7	69.2	57.9	n/a
Voice	69.2	69.2	69.9	**75.2**
Walking	63.2	68.4	70.7	**72.9**
Tapping	60.2	67.7	61.7	**70.7**
	$F_{1}$ ($\%$)			
Memory	70.8	78.8	63.6	n/a
Voice	78.8	75.5	80.6	**83.2**
Walking	69.2	74.7	**77.5**	**77.5**
Tapping	65.4	72.6	72.7	**78.7**

### B. Ensemble Model Performances

#### 1) Classifier Ensemble

In Table IV we show the results of performing the classifier ensemble on the test set within each individual source. Results are divided into two types (i) feature based classifiers only and (ii) feature and CNN based classifiers together. For each, we provide the accuracy and $F_{1}$ for both ensemble algorithms.

**TABLE IV:** Results of the Classifier Ensemble in Each Individual Source When Implemented on the Test Participants. Corresponding Methods: Section II-D1

	LR + RF + DNN		LR + RF + DNN + CNN	
	Majority Voting	Mean Probability	Majority Voting	Mean Probability
	Accuracy ($\%$)	
Memory	69.2	63.2	n/a	n/a
Voice	68.4	71.4	72.9	**78.2**
Walking	69.9	69.2	**77.4**	74.4
Tapping	66.2	63.9	**70.7**	65.4
	$F_{1}$ ($\%$)	
Memory	79.0	70.3	n/a	n/a
Voice	80.6	80.6	82.9	**85.0**
Walking	79.4	75.2	**83.0**	80.0
Tapping	76.9	71.1	**78.9**	71.9

#### 2) Multi-Source Ensemble

In the top portion of Table V, we provide the results of performing a source ensemble (across all four sources) on the test set for each classifier separately. In the bottom portion of Table V we provide the accuracy and $F_{1}$-score resulting from models which combine both classifier and source ensembles.

**TABLE V:** Classification Performances ($\%$) of the Source Ensembles for Each Individual Classifier and for the Combined Classifier and Source Ensemble. Corresponding Methods: Section II-D2

	Majority Voting		Mean Probability	
	Acc.	$F_{1}$	Acc.	$F_{1}$
LR	75.9	83.2	69.2	78.8
RF	74.4	82.7	75.2	82.0
DNN	69.2	80.0	70.7	76.9
CNN	71.4	82.1	79.7	85.9
LR + RF + DNN	78.9	85.3	76.7	82.9
LR + RF + DNN + CNN	82.0	87.1	82.0	87.1

### C. Model and Feature Selection Comparisons

**Fig. 4.**
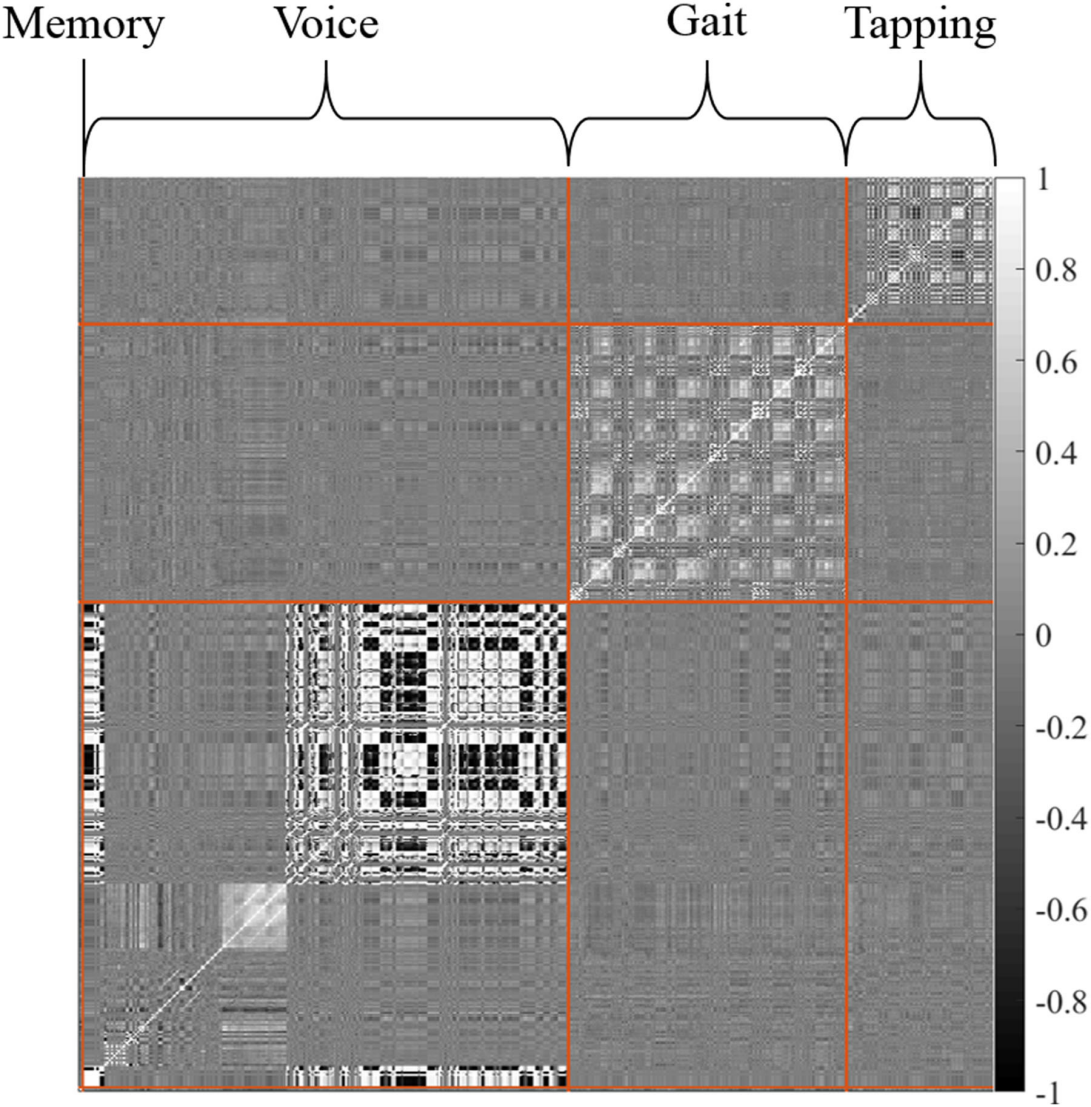
A 606 × 606 correlation matrix showing the inter- and intra-source feature Spearman's Rho correlations using the 133 participants with complete source data. M: Memory, V: Voice, G: Gait, and T: Tapping.

Table VI provides the classification accuracy of the Complete Dataset Learning and Incomplete Dataset Learning techniques. Recall that in Complete Dataset Learning, only the participants with complete source data have been used for selecting features to be utilised during model development. There are $N_{c} =$ 133 participants with complete source data and a total of $p =$ 606 features across the four sources thus $\mathbf {X_{c}} \in \mathbb {R}^{606 \times 133}$. Fig. 4 demonstrates the inter- and intra-source feature correlations of $\mathbf {X}_{c}$. Conversely, recall that Incomplete Dataset Learning entails learning features from participants with missing source data and applying these features to the participants with complete source data. During Incomplete Dataset Learning, the dimensions of the design matrices undergoing LASSO in Equation 1 for the tapping, walking, voice, and memory sources are $\mathbf {X^{T}} \in \mathbb {R}^{97 \times 1323}$, $\mathbf {X^{W}} \in \mathbb {R}^{180 \times 624}$, $\mathbf {X^{V}} \in \mathbb {R}^{326 \times 1072}$, and $\mathbf {X^{M}} \in \mathbb {R}^{3 \times 39}$ respectively.

**TABLE VI:** The Comparison of the Classification Accuracy (%) of the Three Approaches. Corresponding Methods: Section II-E

	LASSO	SG-LASSO
Complete Dataset Learning	71.4 $\pm$ 1.2	73.1 $\pm$ 3.3
Incomplete Dataset Learning	75.6 $\pm$ 1.3	73.2 $\pm$ 1.1
	LR + RF + DNN	LR + RF + DNN + CNN
Multi-Source Ensemble Learning	78.9	82.0

**Fig. 5.**
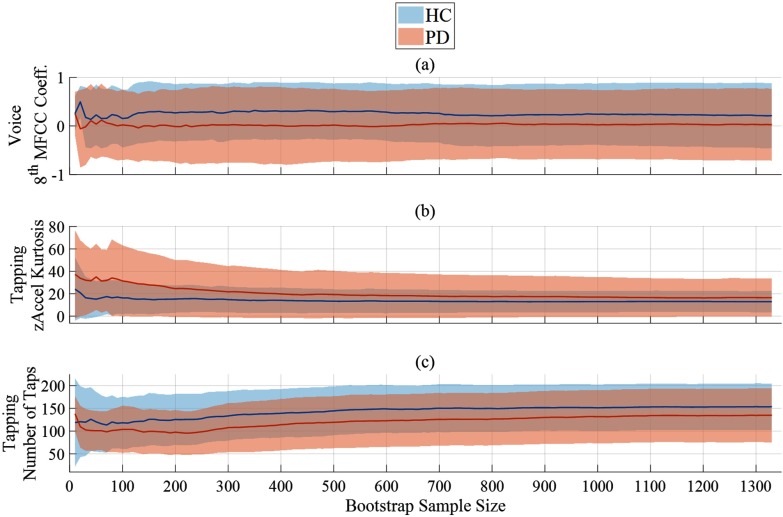
Demonstration of how three feature distributions vary with sample size. There are 10,000 bootstrap samples taken at each sample size. The solid line shows the mean of the mean of each bootstrap sample. The shaded area show the mean standard deviation of each bootstrap sample. Corresponding methods: Section II-F.

Table VII provides the 10 features with the highest weights as determined via LASSO and SG-LASSO during the Complete Dataset Learning and the Incomplete Dataset Learning techniques. Supporting Document 1 provides an exhaustive description of each feature in Table VII. Finally, Fig. 5 shows how three features from the voice, walking, and tapping activities respectively vary with sample size.

**TABLE VII:** The Top 10 Features Selected by the Different Feature Selection Approaches. Recall That Complete Dataset Learning Uses a Small Number Participants With Complete Source Data, Whereas Incomplete Dataset Learning Uses a Large Number of Participants With Incomplete Source Data. Corresponding Methods: Section II-E. T: Tapping, W: Walking, V: Voice, M: Memory. The Feature Names and Definitions are Provided in Supporting Document 1

	Complete Dataset Learning				Incomplete Dataset Learning			
	LASSO		SG-LASSO		LASSO		SG-LASSO	
Feature Rank	Source	Feature	Source	Feature	Source	Feature	Source	Feature
1	V	std-MFCC-10th coef	W	xGyro-DFA	V	std-8th delta	W	xGyro-Mean
2	W	yGyro-Skewness	V	Shimmer-F0-rc75	V	std-MFCC-10th coef	W	xGyro-DFA
3	W	xGyro-Mean	W	zAccel-Median	T	zAccel-Kurtosis	T	zAccel-Median
4	T	xAccel-STD	V	mean-MFCC-8th coef	W	xGyro-Mean	V	mean-11th delta
5	V	std-8th delta	T	zAccel-Skewness	W	xGyro-DFA	W	yAccel-DFA-STD
6	V	GNE-NSR-TKEO	W	yAccel-Kurtosis-STD	V	HNR-mean	V	mean-MFCC-8th coef
7	W	xGyro-DFA	T	zAccel-AR-Lag-1	V	GNE-SNR-TKEO	W	zGyro-Median-STD
8	W	xAccel-Skewness	V	mean-delta delta 0th	V	Shimmer-F0-prc5	W	zAccel-Kurtosis
9	V	Shimmer-F0mean	T	IQR of Tap Distance	W	yGyro-DFA	V	mean-MFCC-5th coef
10	V	Shimmer-F0prc75	V	mean-12th delta-delta	V	Shimmer-F0-FM	W	zGyro-AR-Lag-1-STD

## IV. Discussion and Conclusion

Common techniques for analyzing datasets with large quantities of missing data often result in a significantly smaller subset of the data being analyzed. In this research we have presented a novel method for compensating for source-wise missing data through the combined use of dataset deconstruction and ensemble learning. Our approach ensures a 100% participant retention rate without the need to perform imputation. Unlike previous work, our method identifies a consistent set of participants against which all models can be tested making the results highly interpretable. Due to the inclusion of a high number of participants and the robust fusion of multiple classification models, we find our method yields higher disease classification accuracies when used for remote detection of PD and to also be more appropriate at feature selection than traditional methods.

We developed 12 feature based classifiers and three CNN classifiers. Four of the feature based classifiers utilised a state-of-the-art DNN. Interestingly, we find that the DNN often gives consistent accuracies with the traditional feature based classifiers. This is indicative that the feature set is the limiting factor during classification as opposed to the classification technique. In the three sources where CNNs were developed, the CNN consistently outperformed the traditional feature based classifiers on both the training and test participants. The improvement seen by using CNNs is attributed to the translationally invariant nature of the filters identified from a large number of training instances. As the training sample size is large for all CNNs we can be confident that the resulting filters are robust at a population level and less likely to cause overfitting than in the traditional feature based approaches. This is the first study to the authors knowledge to implement a consistent CNN architecture on multiple types of smartphone sensor data for the purpose of PD classification. These findings are consistent with the hypothesis that more sophisticated machine learning models used in tandem with a large cohort will improve remote PD classification [5].

The purpose of applying a classifier ensemble was to account for variability between classifiers thus providing additional means for accounting for the noise and uncertainty that is inherent in the mPower dataset. The initial classifier ensemble made use of only the feature based classifiers. Although the ensemble often causes a small increase in performance within each separate source, the feature based classifier ensemble often fell short of the classification accuracy achieved by the respective source's CNN for all ensemble techniques. The tapping activity benefits the least from the use of classifier ensembles. Indeed, the highest accuracy of the tapping classifier ensemble is equal to that of the standalone CNN accuracy, with the mean probability ensemble performing worse than the stand alone CNN. This result is attributed to conflicting classifier responses alongside classifier uncertainty - wherein the soft responses of each classifier is close to 0.5. The second ensemble made use of the feature based classifiers and the CNN classifier and in many cases reported accuracies higher than all base classifiers, including the respective source's CNN. Once more this is suggestive that the CNN is correctly classifying different subjects to the feature based classifiers on account of it using the convolutional based filters for classification as opposed to the hand-crafted feature set.

The benefit of the source ensemble approach is far more apparent than that of the classifier ensembles. When using a single classifier from multiple sources, the source ensemble outperforms the majority of single source classifiers. The cause of the improvement seen in the source ensemble approach is two-fold: (i) it accounts for the noise and variation within each of individual source models and (ii) it accounts for participants showing symptom heterogeneity wherein symptoms are only present in some sources.

The final ensemble approach demonstrated that the combined affect of source and classifier ensembles outperform all other ensemble approaches. We again find that ensembles at the source and classifier level show the highest classification accuracy when using both feature and CNN based classifiers. This finding is intuitive given that the classifier and source ensemble, when using the feature and CNN based classifiers, are accounting for the multiple types of noise and variability.

To test the efficacy of multi-source ensemble learning, we performed two types of comparative studies. We firstly implemented Complete Dataset Learning, the most common approach for datasets where source-wise missing data occurs. Here, models that are trained and validated on the participants with complete data showed an increase in classification accuracy when compared to single source models. We also note that of the 1,513 participants used in this research, only 133 (8.8%) had complete source data. As such, when we employ Complete Dataset Learning we are discarding 91.2% of participants. This is the standard approach used in the literature and is clearly a highly inefficient use of data [20], [40]. The second comparative study entailed an inspection on the influence of feature selection. During Incomplete Dataset Learning we performed feature selection within each source individually using the large number of participants with incomplete data and applied these features to the participants with complete data for classification. A single neuron model (LR) was used throughout the comparative studies as a means of assessing the feature selection capabilities of both methods. If more complex models, such as a random forest or a DNN, the classification accuracy may be modified by the inherent capability of these classifiers to perform feature selection. We found that this feature selection approach yielded higher classification accuracy than the Complete Dataset Learning Approach.

The inter-source feature relationship was inspected in several manners. Fig. 4 demonstrates the features between sources show very little correlation although within individual sources correlations exist. This lack of inter-source feature correlation explains why the use of SG-LASSO yields very similar results to that of traditional LASSO. Indeed, during the optimization of Equation 8, the value of $ \lambda _{2}$ tended to be very close to zero. This indicates that regularization occurs almost entirely at the feature level, and is virtually non-existent at the source level. This finding is further demonstrated by the similarity between features selected during the LASSO and SG-LASSO techniques in Table VII. The top ten features selected by the LASSO and SG-LASSO approaches (Table VII) show a high degree of overlap for both Complete and Incomplete Dataset Learning which further confirms the lack of inter-source feature correlations. It is also interesting to note that no features from the memory activity were in the top 10 highest weighted features in any approach. Indeed, only ‘Total Memory Score’ was selected by all approaches but received very low weightings in the feature selection process.

Finally, we explored how sample size affects feature distributions, and consequently how sample size affects the confidence of feature selection. In Fig. 5 it can be seen that feature values at small sample sizes are often transient, showing variable behaviour that is not representative of the population. However, with larger sample sizes the feature distributions reach a pseudo-steady state and show little variation. Fig. 5(a) is an example of a voice feature that is selected by both the Incomplete Dataset Learning and the Complete Dataset Learning processes (Table VII). At sample sizes above 100 the feature distributions are stable and consistently differ between the disease groups. Fig. 5(b) is an example of a tapping feature that is selected during Complete Dataset Learning, but not during Incomplete Dataset Learning. It is evident that at small sample sizes the feature values between the two groups appear to be large, but with the inclusion of more samples this difference is greatly reduced. Finally, in Fig. 5(c) we show the behaviour of the common and interpretable ‘Number of Taps’ feature from the tapping activity. Not only does this feature consistently show a difference between the disease groups, but it shows a gradual change of mean feature value. We attribute the variation of these feature distributions with sample size to high levels of noise in the feature set. Via the inclusion of more participants, our bootstrap estimates are less susceptible to noise and therefore provide better population estimates. As such, we believe the features identified using the large sample size during Incomplete Dataset Learning are more robust and scalable than those identified by Complete Dataset Learning.

A limitation of the presented work is the assumption that all tests to have been completed correctly on the first attempt. Although mostly a correct assumption, we expect that some tests used in this research were entirely noise and contain no relevant information. We intend to alleviate the influence of this limitation via the inclusion of longitudinal test instances. Another limitation is that the efficacy of multi-source ensemble learning on a dataset where inter-source relationships exist was not evaluated. Feature selection at the source level generally improves classification ability where inter-source correlations exist. Our future work intends to further assess multi-source ensemble learning (where feature selection only occurs at the feature level) via implementation on additional datasets wherein inter-source correlations are present. Additional future work will focus on utilising multi-source ensemble learning in creating a disease severity score. This entails applying the method to the longitudinal data and determining whether an objective multi-source composite score can be created that correlates with PD severity. Inclusion of the longitudinal data in the mPower dataset would increase the number of test instances by an order of magnitude; therefore creating an additional and substantial set of data to further test the potential of multi-source ensemble learning.
